# The effect of *MVK-MMAB* variants, their haplotypes and G×E interactions on serum lipid levels and the risk of coronary heart disease and ischemic stroke

**DOI:** 10.18632/oncotarget.20349

**Published:** 2017-08-18

**Authors:** Liu Miao, Rui-Xing Yin, Feng Huang, Wu-Xian Chen, Xiao-Li Cao, Jin-Zhen Wu

**Affiliations:** ^1^ Department of Cardiology, Institute of Cardiovascular Diseases, The First Affiliated Hospital, Guangxi Medical University, Nanning 530021, China; ^2^ Department of Neurology, The First Affiliated Hospital, Guangxi Medical University, Nanning 530021, China

**Keywords:** mevalonate kinase, methylmalonic aciduria (cobalamin deficiency) cblB type, single nucleotide polymorphism, coronary heart disease and ischemic stroke, lipids and interaction

## Abstract

**Aim:**

This study aimed to detect the association of the mevalonate kinase (MVK) and methylmalonic aciduria (cobalamin deficiency) cblB type (MMAB) gene variants, their haplotypes, and gene-environment (G×E) interactions on serum lipid levels and the risk of coronary heart disease (CHD) and ischemic stroke (IS) in a Chinese Han population.

**Methods:**

Genotyping of the rs3759387, rs7134594, rs877710 and rs9593 SNPs in 846 CHD and 869 IS patients and 847 healthy controls was performed by PCR-RFLP and Sanger sequencing. Logistic regression and factor regression were used to investigate the association of 4 *MVK-MMAB* SNPs and serum lipid levels and the risk of CHD and IS.

**Results:**

The genotypic and allelic frequencies of the rs3759387 and rs7134594 SNPs differed between controls and patients (*P* < 0.0125-0.001). The rs3759387 SNP was associated with the risk of CHD and IS in different genetic models. The A-T-G-A and C-T-C-T haplotypes were associated with increased risk of CHD. The haplotype of A-T-G-A was associated with an increased risk of IS, whereas the C-T-G-A haplotype was associated with a decreased risk of IS. Interactions of C-T-C-T-smoking or C-T-C-T-age on the risk of CHD, and A-T-G-A-hypertension or A-T-G-A-age on the risk of IS were also observed. The subjects with the rs3759387AA genotype in controls had lower high-density lipoprotein cholesterol (HDL-C) levels than did the subjects with AC/CC genotypes. Several SNPs interacted with alcohol consumption and cigarette smoking to increase serum HDL-C and apolipoprotein A1 levels, but they interacted with body mass index ≥ 24 kg/m^2^ to decrease serum HDL-C and apolipoprotein A1 levels.

**Conclusion:**

Several *MVK-MMAB* variants, especially the rs3759387 SNP, 4 main haplotypes, and G×E interactions were associated with serum lipid levels and the risk of CHD and IS in a Chinese Han population.

## INTRODUCTION

Coronary heart disease (CHD) and ischemic stroke (IS) are the most prevalent geriatric diseases and the major determinant of mortality and morbidity worldwide [1–3]. More than 700,000 people die from CHD each year in China [4]. As a complex and multifactorial disorder, CHD and IS are resulted from lots of pathogenic factors, including genetic factors and environmental exposures [5]. The major pathological basis of these two diseases had proved to be atherosclerosis which the essential as an ambitious inflammatory disorder. Therefore, both of diseases would be participated in the same genetic and environmental backgrounds, including gender, time to life, hypercholesterol, hypertension, diabetes, cigarette smoking, and genetic factors [6–9]. A large number of genes and loci related to CHD [10] or IS [11] were reported in previous genome-wide association studies (GWASes). In addition, some genetic variants that were initially shown to have an effect on the risk of CHD were detected to be related to IS soon afterwards [12, 13].

Recent GWASes have found several novel loci at chromosome 12q24, including the mevalonate kinase (*MVK*) and methylmalonic aciduria (cobalamin deficiency) cblB type (*MMAB*) genes, both of which influence high-density lipoprotein cholesterol (HDL-C) levels [14, 15]. According to the relevant studies that this region can regulate serum lipid concentrations. [16–18]. Particularly, two head to head genes, *MVK* and *MMAB* took part in metabolic pathways may adjust to HDL metabolism [19]. MVK, encoded by *MVK*, play an important role in an initial stage in cholesterol biosynthesis. In humans, when *MVK* mutations in homozygosity can give rise to hyperimmunoglobulinemia D syndrome, which the basic symptoms were fever and high concentrations of immunoglobulins D and A in blood. When the patients suffered from hyperimmunoglobulinemia D syndrome, low HDL-C levels can be found, in accordance with the latest GWASes findings [14, 15]. However, when somebody lacked of cob (I) alamin adenosyltransferase, as an enzyme encoded by *MMAB*, may contribute to methylmalonic aciduria [20]. But, the exact metabolism by which *MMAB* influences cholesterol is still unknown. A relevant report about schizophrenia had revealed that urinary methylmalonic acid may negatively correlated with red blood cell membrane cholesterol levels in blood [21]. *MVK*, which is close to *MMAB*, gives rise to the increased sensibility of obesity, diabetes and atherosclerosis [22]. Therefore, *MVK* and *MMAB* may be appropriate candidates as genes to elevate HDL-C concentrations and then affect the risk of CHD and IS. Sun *et al.* had taken several SNPs into consideration, including *MVK* (rs3759387 and rs2287218) and *MMAB* (rs12817689, rs22411201, rs11067227, rs7134594, rs877710, rs11067233, rs9593, rs11831226 and rs8228), but only rs11067233 in *MMAB* may contribute to the susceptibility of CHD by decreasing plasma HDL-C levels in Han Chinese [23]. Junyent et *al.* suggested that the *MMAB*-3U3527G/C variant might contribute to the variation in HDL-C concentrations, particularly in subjects with high carbohydrate intakes [24]. In the current study, we aimed to detected whether four SNPs, rs3759387, rs7134594, rs877710 and rs9593 in the *MVK* and *MMAB* and their mutual effect between gene and environment, make an interaction with the risk of CHD and IS in Han populations where located in southern China.

## RESULTS

### General features of the demography

The general trait of the demography is summarized in Table 1. The levels of systolic blood pressure (SBP), pulse pressure, apolipoprotein (Apo) B, and the percentages of hypertension were higher but the concentrations of total cholesterol (TC), HDL-C, low-density lipoprotein cholesterol (LDL-C), the percentages of individuals who consumed alcohol and the ApoA1 to ApoB ratio were lower in persons who suffered from CHD than in normals (*P* < 0.05 for all). The same situation was also found in IS subjects compared to normals (*P* < 0.05 for all).

**Table 1:** Comparison of general characteristics and serum lipid levels between controls and patients

Parameter	Control	CHD	IS	*P_1_*	*P_2_*
Number	847	846	869	-	-
Male/female	607/240	625/221	628/241	0.224	0.267
Age, years^1^	61.71±11.82	62.18±10.58	62.72±12.35	0.423	0.676
Body mass index, kg/m^2^	22.58±3.16	23.89±3.27	23.21±3.39	0.384	0.221
Systolic blood pressure, mmHg	127.96±19.96	133.40±23.27	147.05±21.16	0.000	0.013
Diastolic blood pressure, mmHg	81.86±13.83	79.45±14.33	83.51±13.05	0.073	0.065
Pulse pressure, mmHg	47.48±19.29	57.03±17.36	63.54±17.71	0.000	0.000
Cigarette smoking, n (%)^2^	330 (39.0)	396 (46.8)	379 (43.6)	0.205	0.454
Alcohol consumption, n (%)	354 (41.8)	225 (27.0)	262 (30.1)	0.000	0.000
Total cholesterol, mmol/L	4.91±0.99	4.54±1.26	4.51±1.12	0.000	0.001
Triglyceride, mmol/L^3^	1.36 (0.73)	1.38 (0.95)	1.47 (0.91)	0.642	0.155
HDL-C, mmol/L	1.87±0.48	1.16±0.39	1.23±0.38	0.000	0.000
LDL-C, mmol/L	2.75±0.78	2.71±1.04	2.67±0.91	0.000	0.000
Apolipoprotein (Apo) A1, g/L	1.40±0.26	1.05±0.60	1.01±0.22	0.070	0.061
ApoB, g/L	0.90±0.20	0.91±0.27	0.92±0.25	0.000	0.000
ApoA1/ApoB	1.63±0.53	1.36±2.48	1.26±0.65	0.019	0.000
Diabetes mellitus, n (%)	153 (18.1)	137 (16.2)	159 (18.3)	0.324	0.869
Hypertension, n (%)	310 (36.6)	354 (41.8)	585 (67.3)	0.000	0.000

*CHD*, coronary heart disease; *IS*, ischemic stroke; *HDL-C*, high-density lipoprotein cholesterol; *LDL-C*, low-density lipoprotein cholesterol. ^1^ Mean ± SD determined by *t*-test. ^2^The rate or constituent ratio between the two groups was analyzed by the chi-square test. ^3^Median (interquartile range) tested by the *Wilcoxon-Mann-Whitney* test. *P_1_*, CHD *vs*. controls; *P_2_*, IS *vs*. controls.

### Frequency of genotypes and alleles

**Figure 1:**
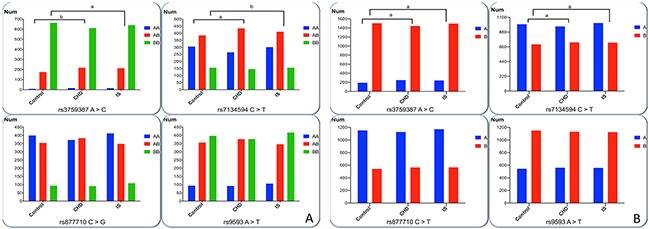
Genotypic and allelic frequencies of four SNPs in controls and patients **(A)** AA genotypes represent as rs3759387AA, rs7134594CC, rs877710CC and rs9593AA. AB genotypes represent as rs3759387AC, rs7134594CT, rs877710CG and s9593AT. BB genotypes represent rs3759387CC, rs7134594TT, rs877710GG and rs9593TT. **(B)** The A allele represents rs3759387A, rs7134594C, rs877710C and rs9593A. The B allele represents rs3759387C, rs7134594T, rs877710G and rs9593T. All of the detected SNPs were in Hardy-Weinberg equilibrium (*P* > 0.05). The rs3759387 A >C and rs7134594 C > T allelic and genotypic frequencies were different among the three groups. *^a^*P< 0.0125; *^b^P* < 0.001.

The frequency of genotypes and alleles of the four *MVK-MMAB* SNPs is showed in Figure 1. Four of the detected SNPs were conformed to Hardy-Weinberg equilibrium (*P* > 0.05). The frequency of genotypes and alleles of rs3759387 and rs7134594, but not rs877710 and rs9593 SNPs, was distinguished normals from patients (CHD and IS, *P* < 0.05 for all). The rs3759387A allele and rs3759387AA genotype frequencies were higher in CHD (A, 14.70%; AA, 1.89%) and IS (A, 13.98%; AA, 1.73%) patients than in control subjects (A, 11.20%; AA, 0.95%; *P* < 0.01 for all). The rs7134594T allele and rs7134594CT genotype frequencies were higher in CHD (T, 42.96%; CT, 50.23%) and IS (T, 41.60%; CT, 47.3%) patients than in control subjects (T, 41.14%; CT, 45.45%; *P* < 0.05 for all).

### Genotypes and the risk of diseases

As presented in Table 2, in different genetic models, after Bonferroni correction only genotypes of rs3759387 were associated with the risk of CHD (*P* < 0.0125 for 0.05 adjusted for 4 variables was considered statistically significant). The dominant model: AA/AC *vs*. CC (OR = 1.44, 95% CI = 1.14–1.82, *P* = 0.0023) and log-additive model: A *vs.* C (OR = 1.42, 95% CI = 1.15–1.77, *P* = 0.0042) can be considered as a statistically meaning. Similarly, dominant model: AA/AC *vs.* CC (OR = 1.32, 95% CI = 1.04–1.69, *P* = 0.0018) and log-additive model: A *vs.* C (OR = 1.29, 95% CI = 1.03–1.61, *P* = 0.0025) in genotypes of the rs3759387 SNP were also increased the risk of IS.

**Table 2:** Genotypes of the four *MVK-MMAB* SNPs and the risk of CHD and IS

SNP/Model	Ref. Genotype	Effect Genotype	CHD (OR 95% CI)	*P*	IS (OR 95% CI)	*P*
rs3759387						
Codominant	CC	AC	1.40 (1.10-1.78)	0.053	1.32 (1.03-1.70)	0.077
		AA	2.30 (0.95-5.58)		1.30 (0.51-3.30)	
Dominant	CC	AC/AA	1.44 (1.14-1.82)	0.0023	1.32 (1.04-1.69)	0.0018
Recessive	CC/AC	AA	2.12 (0.88-5.14)	0.087	1.22 (0.48-3.08)	0.68
Overdominant	CC/AA	AC	1.38 (1.08-1.75)	0.056	1.32 (1.03-1.69)	0.028
Log-additive			1.42 (1.15-1.77)	0.0042	1.29 (1.03-1.61)	0.0025
rs7134594						
Codominant	CC	CT	1.32 (1.05-1.65)	0.051	1.05 (0.83-1.32)	0.68
		TT	1.10 (0.83-1.49)		0.92 (0.68-1.24)	
Dominant	CC	CT/ TT	1.26 (1.02-1.56)	0.034	0.81 (1.01-1.26)	0.92
Recessive	CC/ CT	TT	0.94 (0.72-1.22)	0.64	0.90 (0.69-1.17)	0.43
Overdominant	CC/ TT	CT	1.27 (1.04-1.55)	0.019	1.08 (0.88-1.32)	0.48
Log-additive			1.09 (0.94-1.26)	0.24	0.97 (0.84-1.13)	0.72
rs877710						
Codominant	CC	CG	1.17 (0.95-1.45)	0.33	0.90 (0.72-1.12)	0.59
		GG	1.02 (0.73-1.44)		1.02 (0.73-1.43)	
Dominant	CC	CG/GG	1.14 (0.93-1.39)	0.21	0.93 (0.75-1.14)	0.46
Recessive	CC/CG	GG	0.95 (0.69-1.31)	0.74	1.07 (0.78-1.48)	0.66
Overdominant	CC/GG	CG	1.16 (0.95-1.43)	0.14	0.90 (0.73-1.11)	0.30
Log-additive			1.06 (0.91-1.24)	0.43	0.98 (0.84-1.14)	0.75
rs9593						
Codominant	TT	AT	1.13 (0.91-1.39)	0.54	0.88 (0.70-1.09)	0.48
		AA	1.02 (0.73-1.43)		0.98 (0.71-1.37)	
Dominant	TT	AT/AA	1.00 (0.75-1.35)	0.98	0.90 (0.73-1.11)	0.65
Recessive	TT/AT	AA	0.96 (0.70-1.33)	0.82	1.04 (0.76-1.44)	0.79
Overdominant	TT/AA	AT	1.04 (0.77-1.40)	0.82	0.88 (0.71-1.08)	0.23
Log-additive			0.98 (0.74-1.29)	0.86	0.95 (0.82-1.11)	0.54

*SNP*, single nucleotide polymorphism; *CHD*, coronary heart disease; *IS*, ischemic stroke.

### Interactions of the rs3759387 SNP and environmental exposures on the risk of diseases

**Figure 2:**
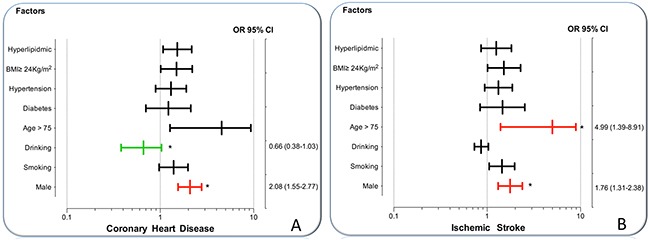
The interactions of the *MVK* rs3759387 SNP and drinking, smoking, BMI, age, hypertension, diabetes, hyperlipidemia and sex on the risk of CHD and IS **(A)** The rs3759387 AA/AC genotypes interacted with male to increase the risk of CHD (OR = 2.08, 95% CI = 1.55 −2.77) and alcohol consumption to decrease the risk of CHD (OR = 0.66, 95% CI = 0.38–1.03). **(B)** The rs3759387 AA/AC genotypes interacted with male to increase the risk of IS (OR = 1.76, 95% CI = 1.31–2.38) and age > 75 years to increase the risk of IS (OR = 4.99, 95% CI = 1.39–8.91). *P < 0.001.

As presented in Figure 2, the rs3759387 AA/AC genotypes interacted with males resulted in increasing the risk of CHD (OR = 2.08, 95% CI = 1.55–2.77, *P* < 0.001) and alcohol drinking to reduce the risk of CHD (OR = 0.66, 95% CI = 0.38–1.03, *P* < 0.001). The rs3759387 AA/AC genotypes also interacted with males (OR = 1.76, 95% CI = 1.31–2.38) and age > 75 years to increase the risk of IS (OR = 4.99, 95% CI = 1.39–8.91, *P* < 0.001).

### Haplotypes and the risk of diseases

Strong LD can be found among the rs3759387, rs7134594, rs877710 and rs9593 SNPs in normals and patients (*D’* = 0.92–0.99) (Supplementary Figure 1). Besides, haplotype analyses which combined with the four SNPs, and the associations of their different haplotypes and the risk of diseases had also been carried out. The four main haplotypes are presented in Table 3. The A-T-G-A and C-T-C-T haplotypes were associated with an increased risk of CHD (adjusted OR = 1.43, 95% CI = 1.14–1.81, *P* = 0.023 and OR = 1.30, 95% CI = 1.01–1.68, *P* = 0.045, respectively). The A-T-G-A haplotype was associated with an increased risk of IS (adjusted OR = 1.28, 95% CI = 1.01–1.63, *P* = 0.041), but the C-T-G-A haplotype was associated with a decreased risk of IS (adjusted OR = 0.83, 95% CI = 0.69–0.99, *P* = 0.043).

**Table 3:** Haplotype frequencies of the four *MVK-MMAB* SNPs and the risk of CHD and IS

Haplotype	Control Frequency	CHD			IS		
		Frequency	OR (95% CI)	*P*	Frequency	OR (95% CI)	*P*
C-C-C-T	0.5840	0.5565	1.00	1.00	0.5822	1.00	1.00
C-T-G-A	0.2103	0.1845	0.92 (0.77 - 1.11)	0.39	0.1835	0.83 (0.69 – 0.99)	0.043
A-T-G-A	0.1045	0.1353	1.43 (1.14 - 1.81)	0.023	0.1835	1.28 (1.01 - 1.63)	0.041
C-T-C-T	0.0980	0.1051	1.30 (1.01 - 1.68)	0.045	0.0915	0.99 (0.760 - 1.29)	0.96

*CHD*, coronary heart disease; *IS*, ischemic stroke. The haplotypes consist of four alleles in the order of rs3759387, rs7134594, rs877710 and rs9593 SNPs.

### The mutual effect between the haplotypes and environmental exposures on the risk of diseases

**Figure 3:**
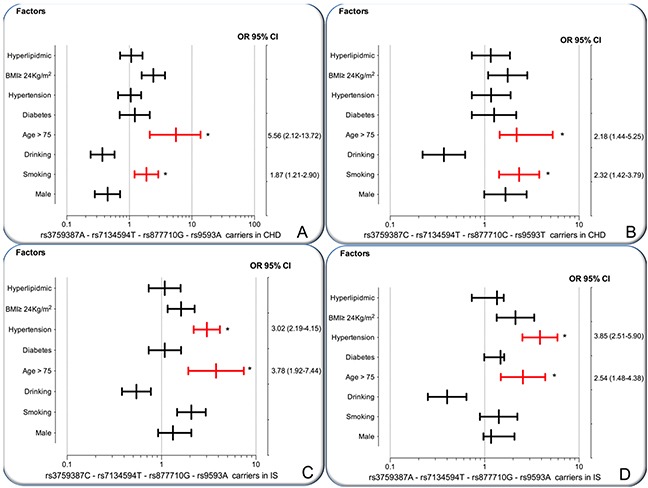
The interactions of the *MVK-MMAB* haplotype and the risk of CHD and IS *CHD*, coronary heart disease; *IS*, ischemic stroke. **(A and B)** The rs3759387A-rs7134594T-rs877710G-rs9593A carriers and rs3759387C-rs7134594T-rs877710C-rs9593T carriers interacted with cigarette smoking and age > 75 years to increase the risk of CHD. **(C and D)** The rs3759387C-rs7134594T-rs877710G-rs9593A carriers and rs3759387A-rs7134594-rs877710G-rs9593A carriers interacted with hypertension and age > 75 years to increase the risk of IS. *P < 0.001.

As shown in Figure 3, the interactions of the *MVK-MMAB* haplotypes and several risk factors for CHD and IS were noted in this study. The interactions of rs3759387A-rs7134594T-rs877710G-rs9593A-smoking (adjusted OR = 1.87, 95% CI = 1.21–2.90, *P* = 0.00022), rs3759387A-rs7134594T-rs877710G-rs9593A-age > 75 years (OR = 5.56, 95% CI = 2.12–13.72, *P* < 0.001), rs3759387C-rs7134594T-rs877710C-rs9593T-smoking (OR = 2.32, 95% CI = 1.42–3.79, *P* = 0.00002), and rs3759387C-rs7134594T-rs877710C-rs9593T-age > 75 years (OR = 2.18, 95% CI = 1.44–5.25, *P* = 0.0016) increased the risk of CHD.

The interactions of rs3759387C-rs7134594T-rs877710G-rs9593A-hypertension (adjusted OR = 3.02, 95% CI = 2.19–4.15, *P* = 0.00012), rs3759387C-rs7134594T-rs877710G-rs9593A-age > 75 years (OR = 3.78, 95% CI = 1.92–7.44, *P* = 0.0009), rs3759387A-rs7134594T-rs877710G-rs9593A-hypertension (OR = 3.85, 95% CI = 2.51–5.90, *P* = 0.0001), and rs3759387A-rs7134594T-rs877710G-rs9593A-age > 75 years (OR = 2.54, 95% CI = 1.48–4.38, *P* < 0.001) increased the risk of IS.

### Genotypes and serum lipid concentrations

**Figure 4:**
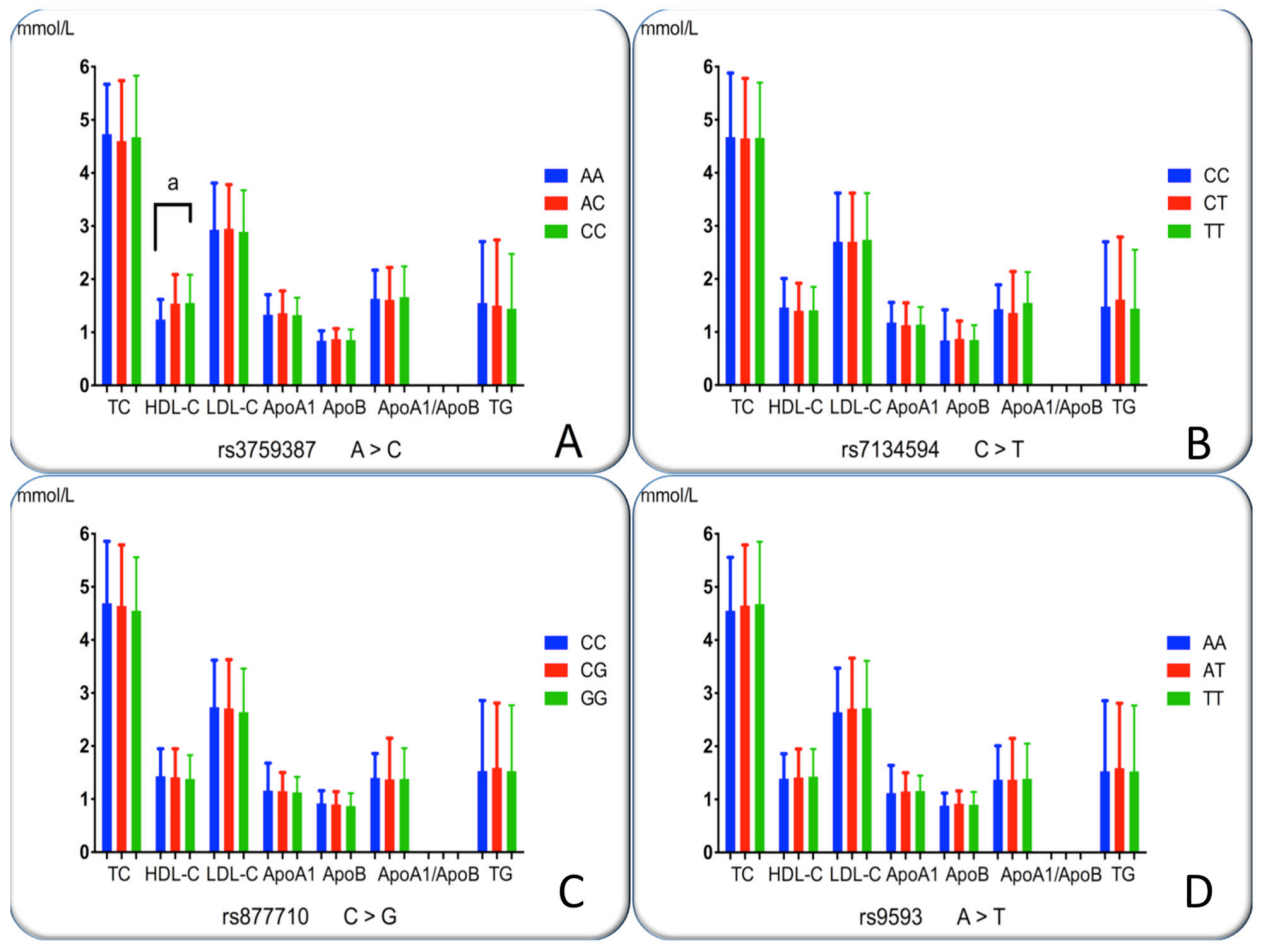
Genotypes of the four *MVK-MMAB* SNPs and serum lipid levels in controls *TC*, total cholesterol; *TG*, triglyceride; *HDL-C*, high-density lipoprotein cholesterol; *LDL-C*, low-density lipoprotein cholesterol; *ApoA1*, apolipoprotein A1; *ApoB*, apolipoprotein B; *ApoA1/ApoB*, the ratio of apolipoprotein A1 to apolipoprotein B. The value of triglyceride is presented as the median (interquartile range), and the difference among the genotypes was determined by the Kruskal-Wallis test. ^a^*P*< 0.0125 (after adjusting for 4 independent tests by the Bonferroni correction). **(A)**, rs3759387; **(B)**, rs7134594; **(C)**, rs877710; and **(D)**, rs9593 SNPs.

The association of the *MVK-MMAB* SNPs and serum lipid concentrations in normals is shown in Figure 4. There were distinct serum HDL-C concentrations among the three genotypes of the rs3759387 (*P* = 0.008); however, no positive findings were observed among the other three SNPs (*P* > 0.0125 for all). The subjects with the rs3759387AA genotype had lower HDL-C concentrations than did the individuals with rs3759387AC and rs3759387CC genotypes. There were no differences in serum TC, triglycerides (TG), LDL-C, ApoA1, ApoB concentrations, and the ratio of ApoA1 to ApoB among the three genotypes of the SNPs.

### The interactions between the *MVK-MMAB* SNPs and several environmental exposures on serum lipid concentrations and the risk of diseases

**Figure 5:**
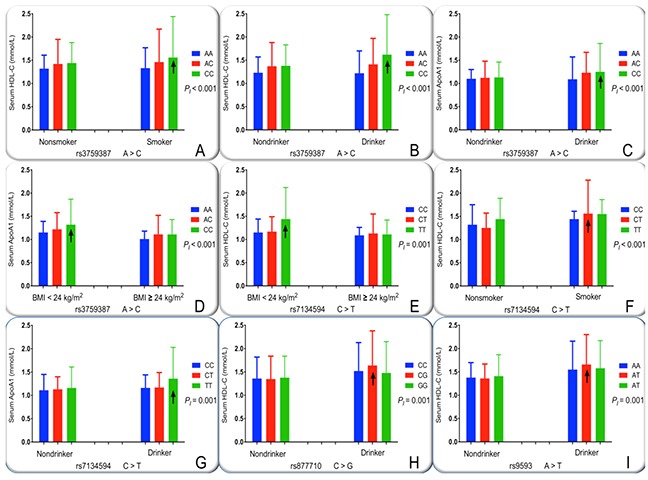
The effect of interactions of the *MVK-MMAB* SNPs and drinking, smoking and BMI on serum lipid levels *HDL-C*, high-density lipoprotein cholesterol; *ApoA1*, apolipoprotein A1; *BMI*, body mass index. The differences in serum HDL-C and ApoA1 levels among the genotypes were assessed using analysis of covariance. The the interactions of the genotypes and alcohol consumption, cigarette smoking or BMI ≥ 24 kg/m^2^ on serum lipid levels were detected by using a factorial regression analysis after controlling for potential confounders (*P*_I_). ↑, genotype and alcohol consumption or cigarette smoking or BMI ≥ 24 kg/m^2^ interaction increased serum lipid levels. *P_I_* ≤ 0.00125 was considered statistically significant after Bonferroni correction (corresponding to *P* < 0.05 after adjusting for five environment exposures multiply eight outcomes by the Bonferroni correction). **(A)**, rs3759387-smoking on HDL-C; **(B)**, rs3759387-drinking on HDL-C; **(C)**, rs3759387-drinking on ApoA1; **(D)**, rs3759387-BMI on ApoA1; **(E)**, rs7134594-BMI on HDL-C; **(F)**, rs7134594-smoking on HDL-C; **(G)**, rs7134594-drinking on ApoA1; **(H)**, rs877710-drinking on HDL-C; and **(I)**, rs9593-drinking on HDL-C.

The interactions between the *MVK-MMAB* SNPs and several environmental exposures, including alcohol consumption, smoking, BMI, age, and sex, on serum lipid levels and the risk of CHD and IS are revealed in Table 4. The mutual effect between some SNPs and alcohol consumption to add serum HDL-C (rs3759387, Figure 5B; rs877710, Figure 5H; and rs9593, Figure 5I) and ApoA1 (rs3759387, Figure 5C; rs7134594 Figure 5G) levels. The mutual effect between rs3759387 (Figure 5A) and rs7134594 (Figure 5F) and cigarette smoking to add serum HDL-C concentrations. The mutual effect between rs7134594 and BMI ≥ 24 kg/m^2^ to reduce serum HDL-C (Figure 5E) concentrations. The mutual effect between rs3759387 and BMI ≥ 24 kg/m^2^ to reduce serum ApoA1 (Figure 5D) concentrations.

**Table 4:** The *P_I_* values for the interactions of genotypes and drinking, smoking, and BMI on serum lipid levels and the risk of CHD and IS

SNP/Factor	Lipid							CHD	IS
	TC	TG	HDL-C	LDL-C	ApoA1	ApoB	ApoA1/ApoB		
rs3759387									
Smoking	0.126	0.447	0.000	0.035	0.029	0.131	0.878	0.616	0.298
Drinking	0.003	0.380	0.000	0.749	0.000	0.016	0.728	0.433	0.193
BMI	0.005	0.293	0.000	0.004	0.003	0.046	0.044	0.276	0.888
Age	0.007	0.013	0.016	0.066	0.005	0.016	0.340	0.245	0.960
Sex	0.011	0.003	0.022	0.072	0.004	0.003	0.198	0.456	0.267
rs7134594									
Smoking	0.994	0.381	0.000	0.176	0.670	0.381	0.004	0.450	0.785
Drinking	0.229	0.239	0.000	0.299	0.001	0.239	0.909	0.507	0.504
BMI	0.040	0.004	0.001	0.003	0.727	0.059	0.081	0.587	0.059
Age	0.785	0.590	0.883	0.702	0.375	0.727	0.112	0.329	0.976
Sex	0.241	0.504	0.694	0.039	0.290	0.241	0.198	0.956	0.096
rs877710									
Smoking	0.727	0.375	0.991	0.035	0.072	0.975	0.909	0.507	0.504
Drinking	0.700	0.618	0.001	0.383	0.404	0.639	0.355	0.906	0.864
BMI	0.264	0.198	0.438	0.702	0.135	0.207	0.507	0.253	0.743
Age	0.869	0.459	0.081	0.262	0.628	0.308	0.016	0.507	0.681
Sex	0.131	0.811	0.142	0.653	0.016	0.011	0.431	0.842	0.502
rs9593									
Smoking	0.709	0.492	0.441	0.081	0.262	0.507	0.253	0.239	0.371
Drinking	0.404	0.633	0.001	0.112	0.702	0.375	0.727	0.004	0.991
BMI	0.135	0.753	0.264	0.198	0.438	0.089	0.752	0.590	0.672
Age	0.628	0.782	0.066	0.348	0.653	0.565	0.290	0.565	0.587
Sex	0.354	0.851	0.445	0.195	0.842	0.032	0.792	0.693	0.814

*SNP*, single nucleotide polymorphism; *TC*, total cholesterol; *TG*, triglyceride; *HDL-C*, high-density lipoprotein cholesterol; *LDL-C*, low-density lipoprotein cholesterol; *ApoA1*, apolipoprotein A1; *ApoB*, apolipoprotein B; *CHD*, coronary heart disease; *IS*, ischemic stroke; *BMI*, body mass index. *P_I_* ≤ 0.00125 was considered statistically significant after Bonferroni correction.

### Relative factors for serum lipid parameters

**Figure 6:**
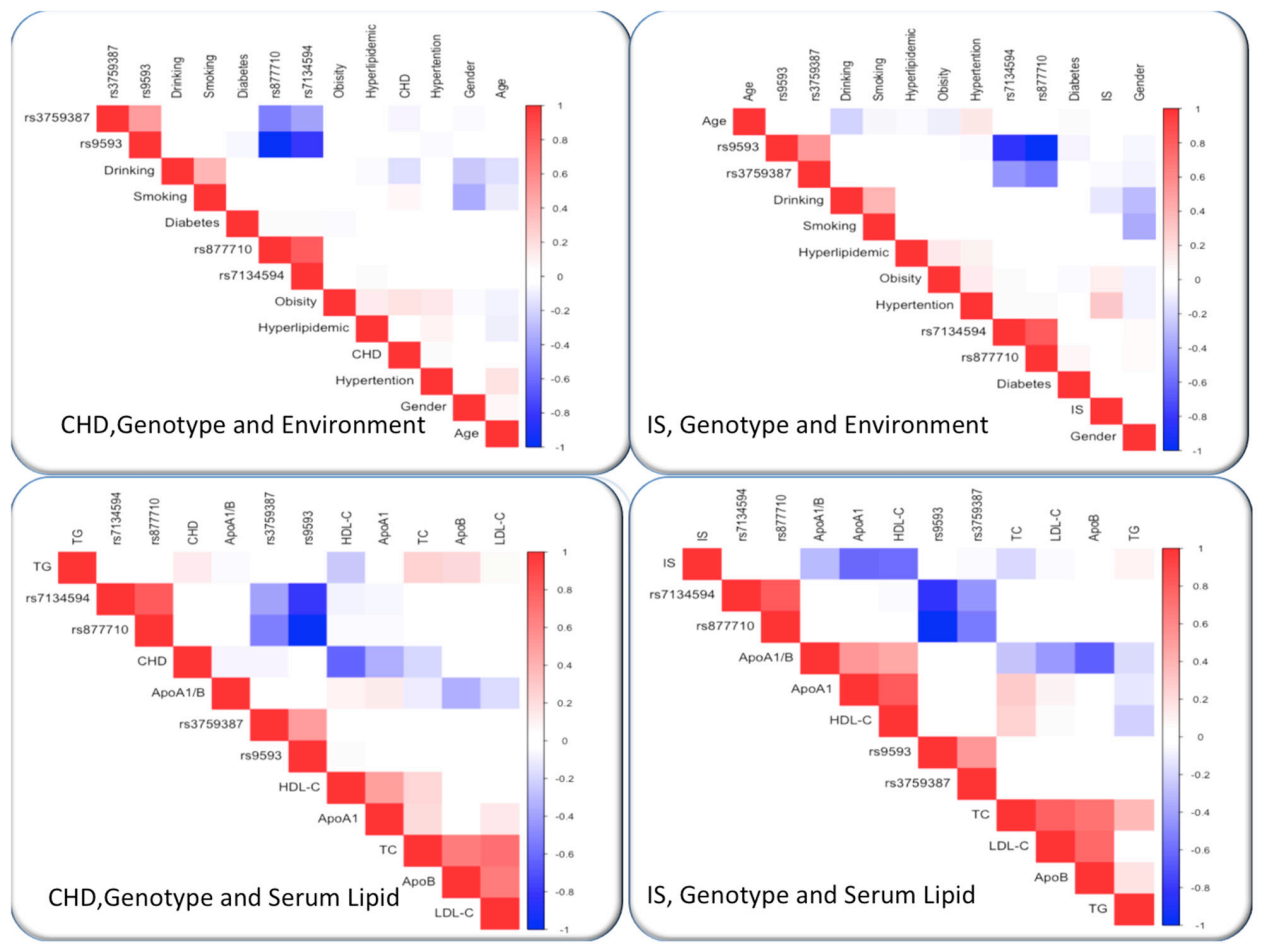
Correlation among environmental exposures and serum lipid variables, as well as the candidate loci in CHD or IS *CHD*, coronary heart disease; *IS*, ischemic stroke. *TC*, total cholesterol; *TG*, triglyceride; *HDL-C*, high-density lipoprotein cholesterol; *LDL-C*, low-density lipoprotein cholesterol; *ApoA1*, apolipoprotein A1; *ApoB*, apolipoprotein B; *ApoA1/B*, the ratio of apolipoprotein A1 to apolipoprotein B; *BMI*, body mass index.

As shown in Figure 6, Pearson correlation analysis showed that several environmental exposures just as time to life, sex, cigarette smoking, and alcohol consumption and traditional cardiovascular risk factors such as BMI and blood pressure levels, also correlated with serum lipid phenotypes of the patient groups.

## DISCUSSION

In the current study, we showed distinctions between the frequency of genotypes and alleles of the rs3759387 and rs7134594 SNPs between normals and CHD or IS patients. Only the rs3759387 genotypes or alleles were associated with the disease risk. In different genetic model, the mutual effect between dominant genotypes and males to add the risk of CHD, but they interacted with alcohol drinking to reduce the risk of CHD. Similarly, the dominant genotypes interacted with males and age > 75 years to increase the risk of IS. The SNPs of rs3759387, rs7134594, rs877710 and rs9593 were in strong LD in normals and patients. Four main haplotypes were detected among the four SNPs. The haplotypes of A-T-G-A and C-T-C-T were associated with an added risk of CHD, but the haplotypes of C-T-G-A and A-T-G-A were associated with an added risk of IS. The individuals with the rs3759387AA genotype in normals had lower HDL-C concentrations than did the individuals with rs3759387AC/CC genotypes. The mutual effect between several SNPs and alcohol drinking to affect serum HDL-C (rs3759387, rs877710 and rs9593) and ApoA1 (rs3759387 and rs7134594) levels. The mutual effect between rs3759387 and rs7134594 and cigarette smoking to influence serum HDL-C concentrations. Two SNPs interacted with BMI ≥ 24 kg/m^2^ to decrease serum HDL-C (rs7134594) and ApoA1 (rs3759387) levels. The interactions of haplotypes and several risk factors on CHD and IS were also observed. Several environmental exposures and traditional cardiovascular risk factors were also correlated with serum lipid phenotypes in both patient groups. As far as we had known that this is the first report to detect the interactions among the four *MVK-MMAB* SNPs and their haplotypes and environmental exposures on serum lipid concentrations and the risk of CHD and IS.

We revealed that the frequency of rs3759387 and rs7134594 genotypes and alleles were distinct from normals and CHD or IS patients. The patients with CHD or IS had higher frequencies of the rs3759387A allele than did the controls. At the same time, the patients with CHD or IS had higher frequencies of the rs7134594T allele than did the controls. The rs3759387 SNP was also associated with the risk of CHD and IS after Bonferroni correction in different genetic models and the mutual effect between dominant genotypes and environmental exposures to increase the risk of CHD and IS.

In a previous GWAS, the information in the International HapMap Project's database showed that the rs3759387A allelic frequency was 23.3% in Europeans, 17.8% in Han Chinese in Beijing (CHB), 9.9% in Japanese, and 45.1% in Sub-Saharan African. Besides, we also discovered that the rs3759387A allelic frequency in our current research populations was lower than in CHB in comparison with the other populations, a reasonable explanation would be contributed to different sample numbers and because of CHB in Guangxi are a part of Han. Above results remind us that the prevalence of the rs3759387A allele variation may have racial/ethnic specificity. The prevalence of the rs3759387A allele is higher in Europeans than in Chinese. All of these findings would be a reasonable explanation for the distinct prevalence of CHD between European and Chinese.

The association of the rs3759387, rs7134594, rs877710 and rs9593 SNPs and the risk of CHD and IS has never been detected in previous studies. In the current study, we revealed that the rs3759387 SNP may have an association with the three SNPs and influence the risk of CHD and IS. Furthermore, high LD among the four SNPs was also found in controls and patients. Moreover, when we analyzed the haplotypes among the four SNPs, the haplotypes of A-T-G-A and C-T-C-T were associated with an increased risk of CHD, whereas the haplotypes of C-T-G-A and A-T-G-A were associated with an increased risk of IS. However, these results require further investigation in the different populations with bigger sample numbers.

**Figure 7:**
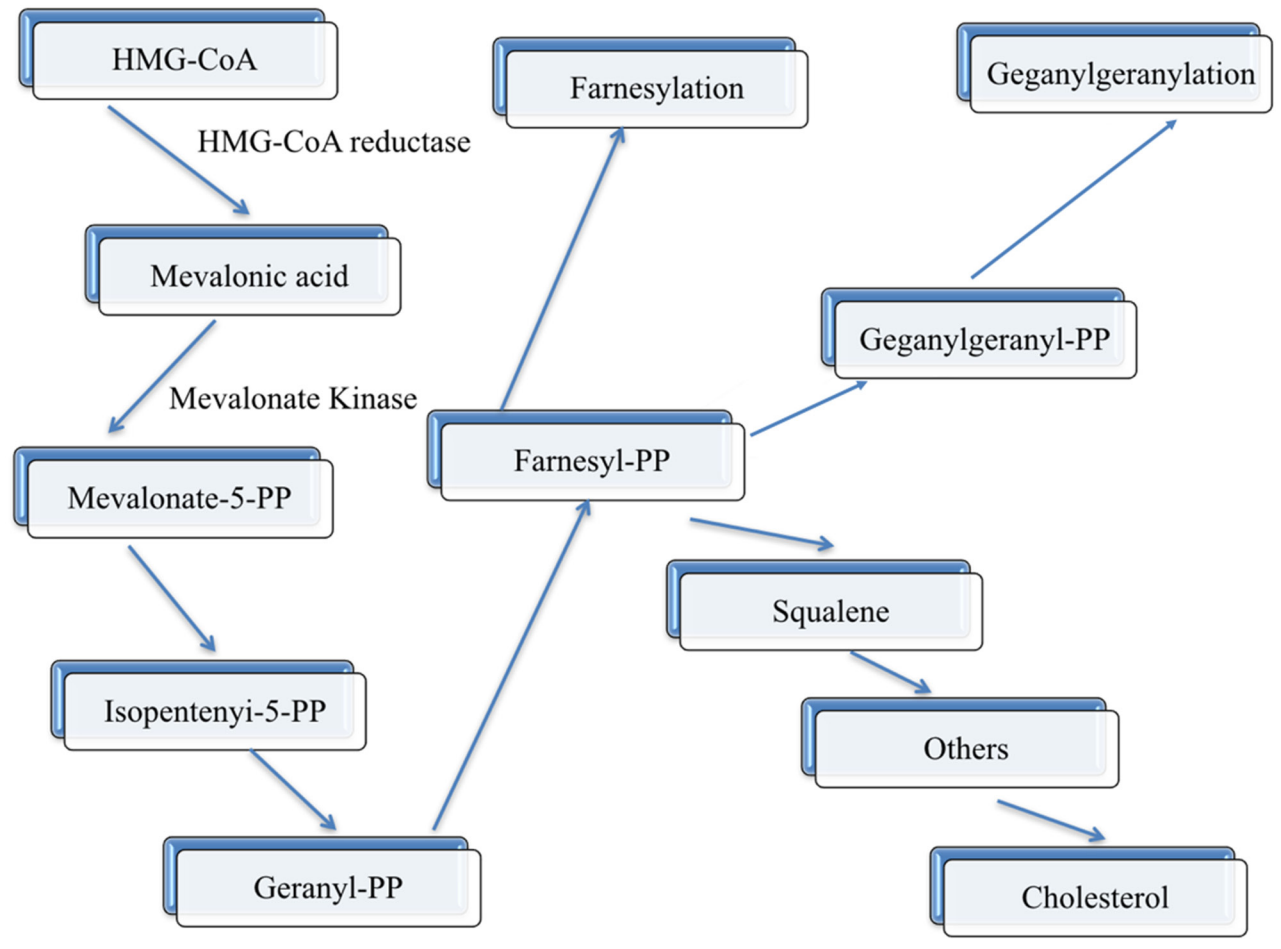
Schematic representation of the mevalonate pathway The enzymes (HMG-CoA reductase and mevalonate kinase) are indicated along the pathway in bold characters.

Previous epidemiological researches have given strong evidence that high serum HDL-C concentrations would be inversed CHD morbidity and mortality [25–27]. Each 1% decrease in LDL-C concentration has been estimated to decrease the risk of CHD by 1% [28], and each 1% increase in HDL-C level decreases the risk of CHD by 2% [29]. The exact mechanism why HDL-C can protection against atherosclerosis includes the below three main points: (1), reverse cholesterol transport from peripheral tissues to the liver [30]; (2), inhibition of LDL-C oxidation; and (3), stabilization of the production of prostacyclin [31]. In humans, *MVK* and *MMAB* are head-to-head orientation and located on chromosome 12. In addition, *MVK* and *MMAB*, which share the same promoter, are both modified by sterol-responsive element-binding protein 2 (*SREBP2*), which is a transcription factor that controls cholesterol homeostasis. What is more, the way in which these two neighboring genes take part in metabolic pathways may have an effect on HDL-C metabolism had been found. *MVK* encodes MVK, play an important role in an initial stage in cholesterol biosynthesis (Figure 7) [32]. In contrast, when lacked of cob (I) alamin adenosyltransferase, as an enzyme encoded by *MMAB*, someone may result to methylmalonic aciduria. The exact reason for *MMAB* adjusted cholesterol metabolism has not been identified, maybe cholesterol synthesis through *SREBP2* can explain our findings [33]. The precise mechanism of *MVK* and *MMAB* on serum lipid metabolism remains to be investigated, which may provide a promising target for medical therapy. Similarly, little is known about the potential mutual effect between the *MVK-MMAB* SNPs and their haplotypes with environmental exposures on serum lipid levels and the risk of CHD and IS. According to the current study, we first reported the mutual effect between some SNPs and alcohol consumption to add serum HDL-C (rs3759387, rs877710 and rs9593) and ApoA1 (rs3759387 and rs7134594) levels. The mutual effect between rs3759387 and rs7134594 and cigarette smoking to add serum HDL-C levels. The mutual effect between rs3759387 and rs7134594 and alcohol consumption to add serum ApoA1 levels. The mutual effect between rs7134594 and BMI ≥ 24 kg/m^2^ to reduce serum HDL-C concentrations.

The rs3759387A-rs7134594T-rs877710G-rs9593A and rs3759387C-rs7134594T-rs877710C-rs9593T haplo-type carriers interacted with cigarette smoking and age > 75 years to increase the risk of CHD. The rs3759387C-rs7134594T-rs877710G-rs9593A and rs3759387A-rs7134594T-rs877710G-rs9593A haplotype carriers interacted with hypertension and age > 75 years to increase the risk of IS.

These findings may reveal a disadvantageous effect of excessive alcohol consumption and cigarette smoking on serum lipid information. The present study has revealed that the mutual effect between rs7134594 CT genotypes and cigarette smoking to add serum HDL-C levels. Additionally, the rs3759387 CC, rs877710 CG and rs9593 AT genotypes interacted with alcohol consumption to increase serum HDL-C levels. Although this is a contradictory finding and the exactly mechanism is unknown, most smokers also get into habit of alcohol consumption, which may be a proper explanation in our study populations, and the potential interactions still need to be further confirmed.

A few potential limitations cannot be ignored. First, compared to many GWASes and replication studies, our sample numbers were relatively small. With these situations, larger sample numbers are needed to determine the consequences in future studies. Significant distinctions from demography were observed between the normal and patient groups. For the sake of statistical analysis accuracy, we adjusted for several environmental exposures, including time to life, sex, BMI, cigarette smoking, and alcohol drinking, but the potential influence of these factors on serum lipid concentrations and the risk of CHD and IS could not be completely eliminated. In addition, because many subjects were taking lipid-lowering drugs treatments, it was not proper to analyze the association of the four SNPs and serum lipid levels in the CHD and IS groups.

Last but not least, an individual's genetic background and various environmental exposures can give rise to both CHD and IS, which are complex multifactorial disorders. The association between four *MVK-MMAB* SNPs and their haplotypes and the risk of CHD and IS had been probed, but many aspects remain to be explored, such as environmental exposures and genetic backgrounds and their mutual effects.

In conclusion, the consequences of the current study showed that the frequency of genotypes and alleles of the rs3759387 and rs7134594 SNPs was distinct from normals and patients. The rs3759387 genotypes were associated with the risk of CHD and IS in different genetic models. The rs3759387 SNP interacted with males to increase the risk of CHD and alcohol consumption to decrease the risk of CHD. The same SNP interacted with males and age > 75 years to increase the risk of IS. Four main haplotypes among the rs3759387, rs7134594, rs877710, and rs9593 SNPs were detected. The A-T-G-A and C-T-C-T haplotypes were associated with an increased risk of CHD. The A-T-G-A haplotype was associated with an increased risk of IS, whereas the C-T-G-A haplotype was associated with a decreased risk of IS. The A-T-G-A and C-T-C-T haplotypes interacted with cigarette smoking and age > 75 years to increase the risk of CHD. The C-T-G-A and A-T-G-A haplotypes interacted with hypertension and age > 75 years to increase the risk of IS. The individuals with the rs3759387AA genotype in normals took effect on reducing HDL-C concentrations than did the subjects with rs3759387AC/CC genotypes. The mutual effect between several SNPs and environmental exposures including alcohol consumption, cigarette smoking, and BMI ≥ 24 kg/m^2^ to adjust serum HDL-C and ApoA1 levels.

## MATERIALS AND METHODS

### Study patients

Totally, 1715 unrelated patients were recruited from the hospitalized patients who were treated in the First Affiliated Hospital, Guangxi Medical University. Among them, 846 subjects suffered from CHD, and another 869 patients were diagnosed with IS. CHD can be defined as including typical ischemic symptoms, plus one or more electrocardiographic changes (ST-segment depression or elevation of ≥ 0.5 mm, T-wave inversion of ≥ 3 mm in ≥ 3 leads, or left bundle branch block), in addition to increases in cardiac markers, such as creatinine kinase-MB and troponin T. Coronary angiography was carried out in patients with CHD. For the independent angiographers, two were blinded to the results of the genotypes. When coronary angiograms were performed, they were observed carefully. A vessel was chosen to be scored, and stenosis ≥ 50% had to be noted in an epicardial coronary vessel of interest or in one of its major branches. In the event of discordance of the number of vessels scored between the two reviewers, angiograms were scored by a third independent reviewer. The CHD subjects could be chosen for to our study when significant coronary stenosis (≥ 50%) was observed in at least one of the three main coronary arteries or their major branches (branch diameter ≥ 2 mm). In addition, the angiographic severity of disease was classified according to the number of coronary vessels with significant stenosis (luminal narrowing ≥ 50%) as one-, two-, or three-vessel disease in the three major coronary arteries [34, 35]. The definition of IS was ensured in accordance with the TOAST (Trial of Org 10172 in Acute Stroke Treatment) criteria [36] after rigorous examination, including neurological test, computed tomography, and/or magnetic resonance imaging (MRI). The IS patients entered in the study included individuals who were eligible for one of the two subtypes of TOAST criteria: large-artery atherosclerosis and small-vessel occlusion. However, if the subjects had a confirmed diagnosis of the below diseases, he/she must be excluded from our study: a history of hematologic or brain MRI revealing cerebral hemorrhage, cardioembolic stroke or unspecified stroke, neoplastic or intracranial space-occupying lesion, infection, other types of intracranial lesions, type 1 diabetes, and renal, liver, thyroid, and autoimmune diseases. IS patients who had a past history of CHD were excluded, as were CHD patients who had a past history of IS.

### Control subjects

When CHD and IS patients were enrolled in our study, 847 control subjects, who were matched by age, gender, and ethnic group, were randomly selected from healthy adults who underwent periodical medical check-ups at the Physical Examination Center of the First Affiliated Hospital, Guangxi Medical University. The controls were healthy, without any CHD and IS details by questionnaires, history-taking, and clinical examination. The examination must be covered lots of items, just as physical examination, blood sampling, electrocardiography, chest X-ray, and Doppler echocardiography. All enrolled individuals were Han Chinese from Guangxi, the People's Republic of China. The relevant information was gathered by trained research staff with standardized questionnaires for all participants, including demography, socioeconomic status, medical history and lifestyle exposures.

This study was carried out following the rules of the Declaration of Helsinki of 1975 (http://www.wma.net/en/30publications/10policies/b3/), revised in 2008. The study design was approved by the Ethics Committee of the First Affiliated Hospital, Guangxi Medical University (No: Lunshen-2011-KY-Guoji-001; Mar. 7, 2011). All procedures were performed in accordance with ethical standards. Informed consent was obtained from all participants before data collection.

### Biochemical measurements

We received venous blood samples from all subjects after at least 12 h of fasting. The enzymatic methods with commercially available kits, including Tcho-1, TG-LH (RANDOX Laboratories Ltd., Ardmore, Diamond Road, Crumlin Co., Antrim, UK, BT29 4QY), Cholestest N HDL, and Cholestest LDL (Daiichi Pure Chemicals Co., Ltd., Tokyo, Japan) were used for detecting levels of serum TC, TG, HDL-C, and LDL-C, respectively, in samples. However, the immunoturbidimetric immunoassay (RANDOX Laboratories Ltd.) was needed to explore serum ApoA1 and ApoB concentrations. All determinations were performed with an autoanalyzer (Type 7170A; Hitachi Ltd., Tokyo, Japan) in the Clinical Science Experiment Center of the First Affiliated Hospital, Guangxi Medical University [37–48].

### Diagnostic criteria

The levels were 3.10–5.17 (TC), 0.56–1.70 (TG), 0.91–1.81 (HDL-C), 2.70–3.20 (LDL-C) mmol/L, 1.00–1.78 (ApoA1), 0.63–1.14 g/L (ApoB), and 1.00–2.50 (ApoA1/B), defined as the normal values [37–49]. According to WHO diagnostic criteria, Type 2 diabetes should be defined as (1) fasting glucose (FPG) ≥ 7.0 mmol/L, (2) 2 h postprandial glucose ≥ 11.1 mmol/L, or (3) self-reported diagnosis of diabetes or use of anti-diabetic medications [50, 51]. Individuals with TC > 5.17 mmol/L and/or TG >1.70 mmol/L were diagnosed as hyperlipidemic [52–54]. The 1999 World Health Organization-International Society of Hypertension Guidelines were used to define the management of hypertension [55–57]. Normal weight, overweight, and obesity were defined as a BMI < 24, 24-28, and > 28 kg/m^2^, respectively [58, 59].

### SNP selection and genotyping

We selected four SNPs in *MVK/MMAB* with the following steps. (1) *MVK* gene clusters, were selected from a previous GWAS associated with lipid-metabolism. *MMAB* gene clusters are found to be close to *MVK* gene clusters and associated with serum lipid levels, especially HDL-C. (2) Tagging SNPs were performed by Haploview (Broad Institute of MIT and Harvard, USA, version 4.2), and functional SNPs were predicted to lead to serum lipid changes from the current version of the online resource (1000 Genome Project Database). (3) SNPs information was obtained from NCBI dbSNP Build 132 (http://www.ncbi.nlm.nih.gov/SNP/). (4) SNPs were restricted to a minor allele frequency (MAF) > 1%. (5) SNPs might be associated with the serum lipid levels or cardiovascular disease in recent studies. (6) *MVK* rs3759587 and rs7134594 and *MMAB* rs9593 and rs877710 were selected by the block-based approach. This strategy was enabled by the correlations between tagging SNPs manifested as LD. Although classic tagging is not the goal of SNP selection, with innovative tagging SNPs selection bias is inevitable. [60–66]. Genomic DNA of the samples was isolated from peripheral blood leukocytes according to the phenol-chloroform method [37–49]. Genotyping of 4 mutations was performed by PCR-RFLP and determined by Sanger sequencing. The characteristics of each mutation and the details of each primer pair, annealing temperature, and length of the PCR products are summarized in Supplementary Table 1 and Supplementary Figures 2 and 3. The PCR products of the samples were sequenced with a sequencer ABI Prism 3100 Genetic Analyzer (Applied Biosystems, International Equipment Trading Ltd., Vernon Hills, IL, USA) at Shanghai Sangon Biological Engineering Technology & Services Co. Ltd., Shanghai China (Supplementary Figure 4).

### Statistical analyses

We employed the statistical software package SPSS 22.0 (SPSS Inc., Chicago, IL, USA) to analyze the data. Quantitative variables are expressed as the means ± standard deviation (because serum TG was not a normal distribution, the levels are presented as medians and interquartile ranges and were analyzed by Wilcoxon-Mann-Whitney test). Qualitative variables are presented as percentages. Allele frequency was determined via direct counting, and the standard goodness-of-fit test was used to test Hardy-Weinberg equilibrium. When analyzing the difference in genotype distribution and sex ratio between the groups, we used a chi-square analysis. The general characteristics between patient and control groups were tested by the Student's unpaired *t*-test. The association of genotypes and serum lipid parameters was tested by analysis of covariance (ANCOVA). Any variants associated with the serum lipid parameter at a value of *P* < 0.0125 (corresponding to *P* < 0.05 after adjusting for four independent tests by the Bonferroni correction) were considered statistically significant. After adjusting for the age, gender, BMI, smoking, and alcohol consumption, we employed unconditional logistic regression to evaluate the correlation between the risk of CHD and IS and genotypes. The same methods were used to calculate the odds ratio (OR) and 95% confidence interval (95% CI). When considering the interactions of four SNPs with environment exposures, including alcohol consumption, cigarette smoking, BMI ≥ 24 kg/m^2^, age, and sex, on serum lipid levels and the risk of CHD and IS, we employed a factorial regression to analysis [67–71] after controlling for potential confounders. A *P*_I_ ≤ 0.00125 was considered statistically significant after Bonferroni correction (corresponding to *P* < 0.05 after adjusting for five environment exposures multiplied by eight outcomes by the Bonferroni correction). Haploview (Broad Institute of MIT and Harvard, USA, version 4.2) analyzed the haplotype frequencies and pairwise LD among the detected SNPs. The heart-map of the inter-locus models was measured by R software (version 3.3.0).

## SUPPLEMENTARY FIGURES AND TABLE


