# Supplementary material for: The effect of MVK-MMAB variants, their haplotypes and G×E interactions on serum lipid levels and the risk of coronary heart disease and ischemic stroke
Source: Oncotarget. 2017 Aug 18;8(42):72801–17. doi: 10.18632/oncotarget.20349 (PMC5641170; doi:10.18632/oncotarget.20349)
Supplement: Supplementary file 1 [file oncotarget-08-72801-s001.pdf]

The effect of *MVK-MMAB* variants, their haplotypes and G×E interactions on serum lipid levels and the risk of coronary heart disease and ischemic stroke

SUPPLEMENTARY MATERIALS

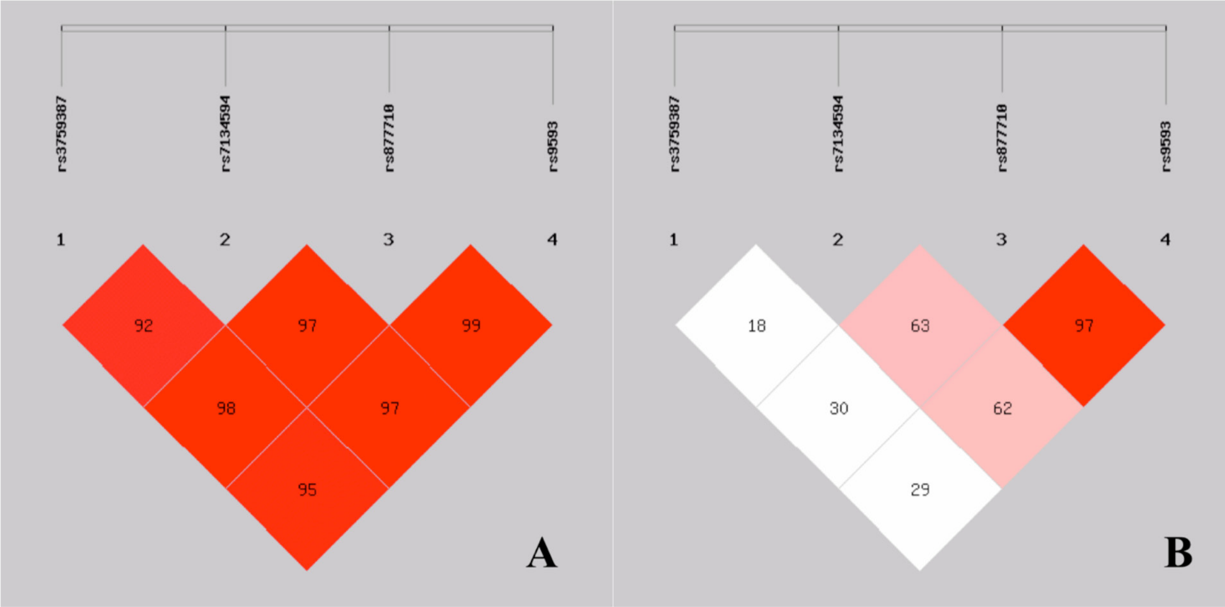

Supplementary Figure 1: The linkage disequilibrium (LD) analyses of the *MVK* rs3759387, *MMAB* rs877710, *MMAB* rs7134594 and *MMAB* rs9593 SNPs in the combined three groups. (A) D'; (B) r^2.

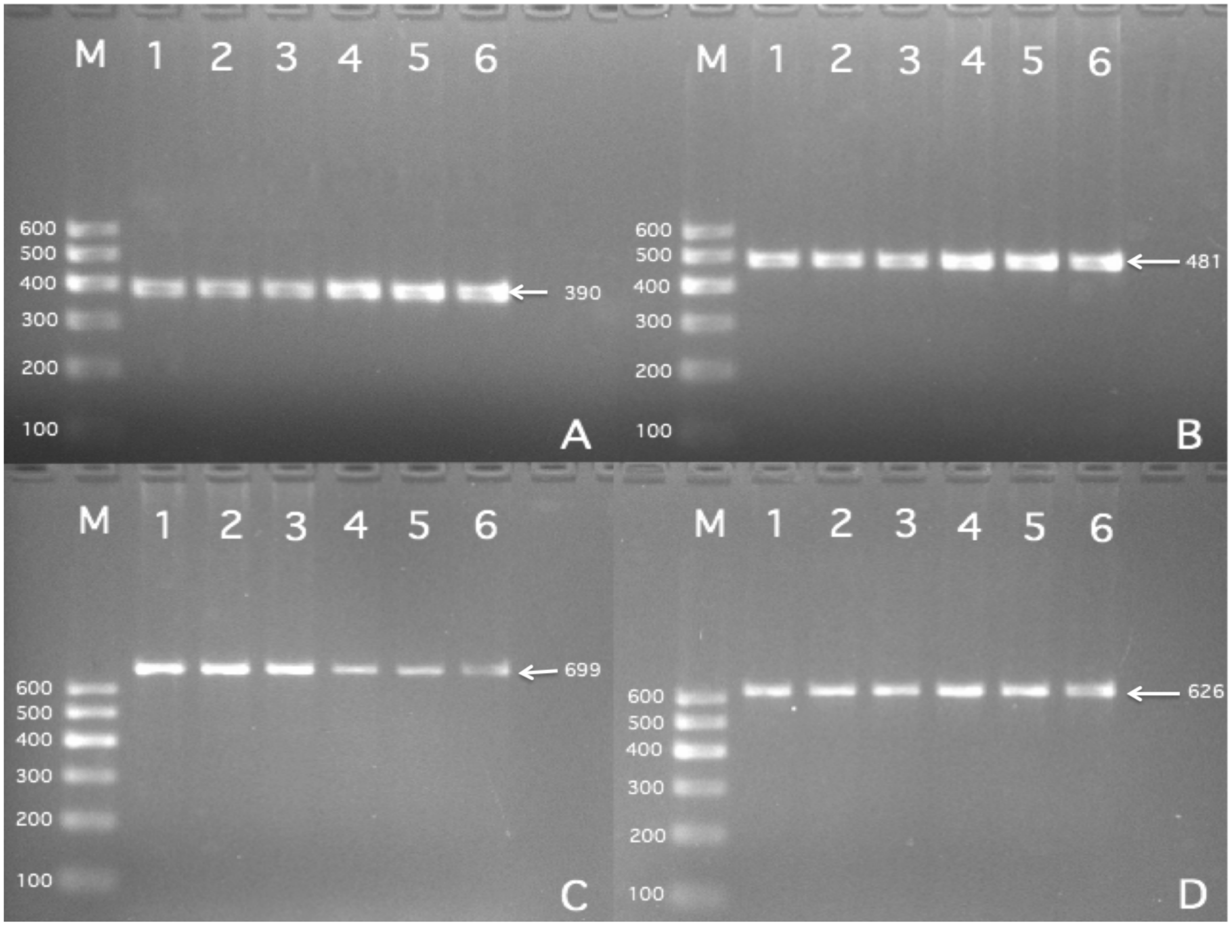

Supplementary Figure 2: Agarose gel electrophoresis (2%) of PCR products of the *MVK* and *MMAB* SNPs. Lane M: DNA ladder 100bp; PCR amplicon of **(A)** *MMAB* rs9593, **(B)** *MMAB* rs7134594, **(C)** *MVK* rs877710, **(D)** *MMAB* rs3759387 SNPs were 390-, 481-, 699- and 626-bp nucleotide sequences; respectively.

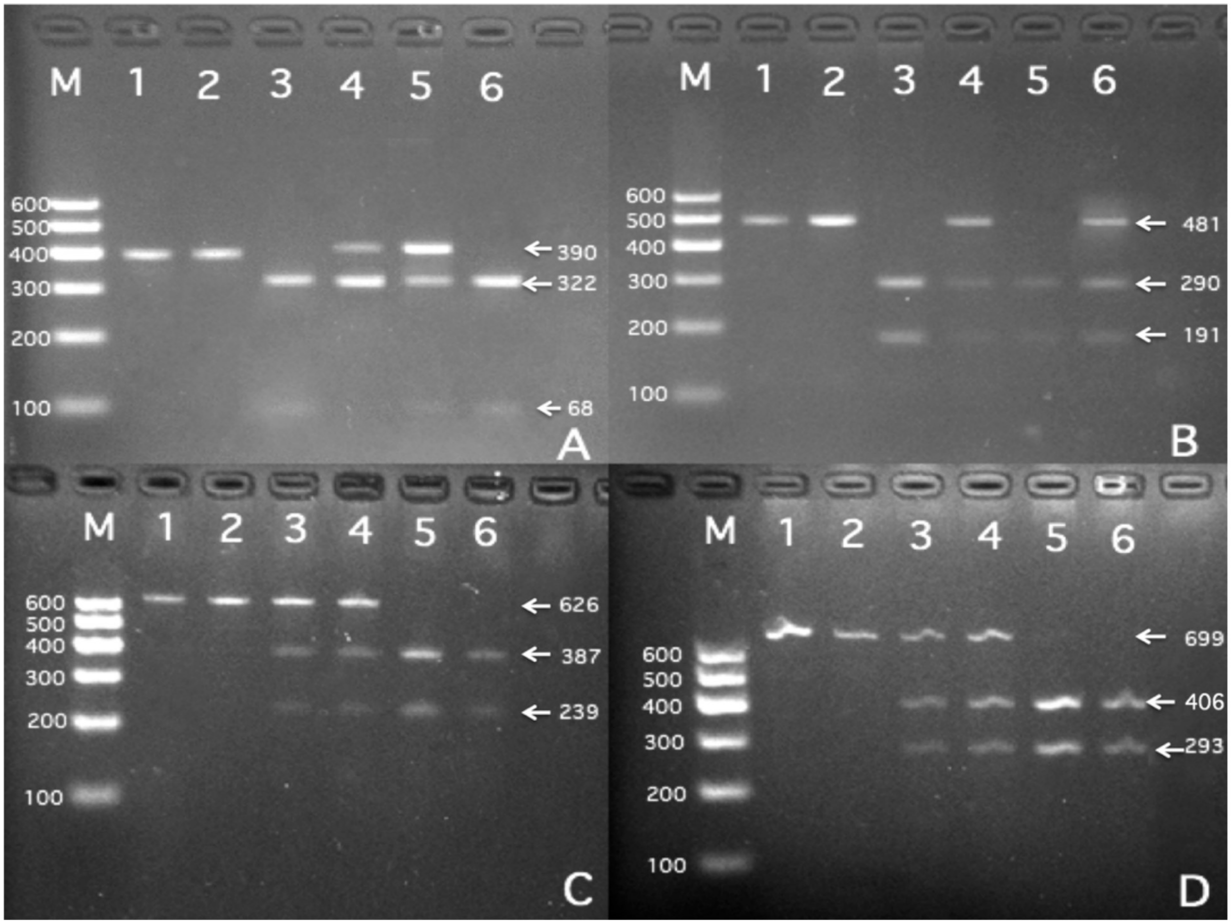

Supplementary Figure 3: Agarose gel electrophoresis (2%) of genotyping of the *MVK* and *MMAB* SNPs. Lane M: DNA ladder100bp. The genotypes of 4 SNPs were as follow: **(A)** *MMAB* rs9593: AA (lanes 1 and 2, 390-bp); AT (lanes 4 and 5, 390-, 322- and 68-bp); and TT genotype (lanes 3 and 6, 322- and 68-bp). **(B)** *MMAB* rs7134594: CC (lanes 1 and 2, 481-bp); CT (lanes 4 and 6, 481-, 290- and 191-bp); and TT genotype (lanes 3 and 5, 290- and 191-bp). **(C)** *MVK* rs3759387: AA (lanes 1 and 2, 626-bp); AC (lanes 3 and 4, 626-, 387-, 239-bp); and CC genotype (lanes 5 and 6, 387- and 239-bp). **(D)** *MMAB* rs877710: CC (lanes 1 and 2, 699- bp); CT (lanes 3 and 4, 699-, 406- and 293-bp); and TT genotype (lanes 5 and 6, 406- and 293-bp).

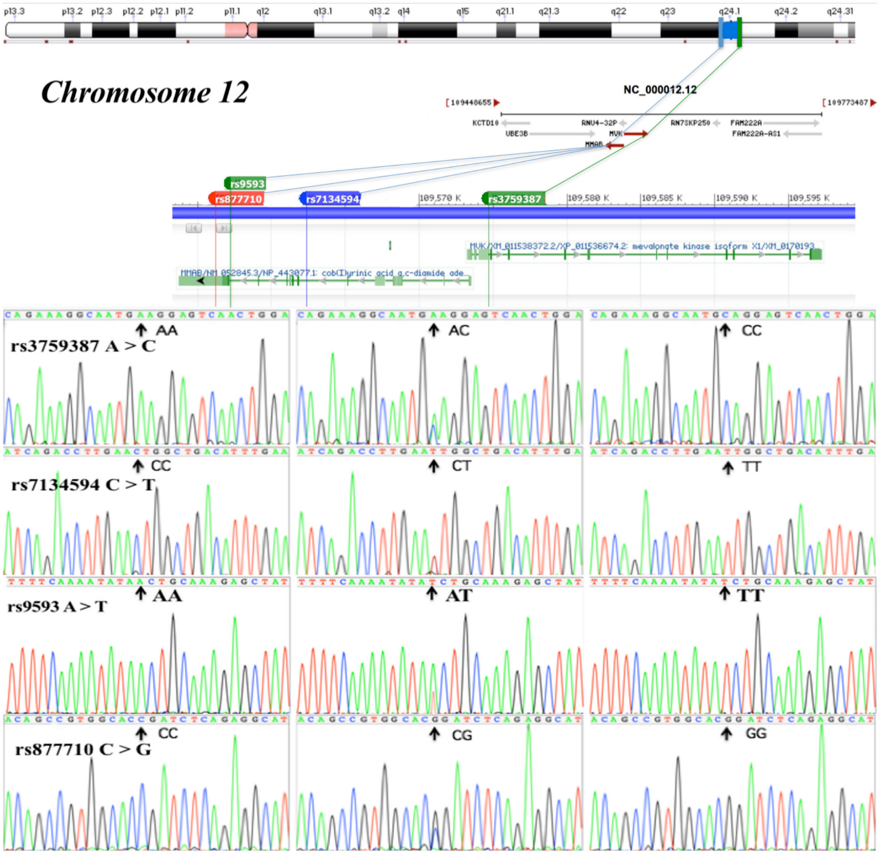

Supplementary Figure 4: The positions and parts of the nucleotide direct sequencing results of the *MVK* and *MMAB* SNPs. *MVK*, mevalonate kinase; *MMAB*, methylmalonic aciduria (cobalamin deficiency) cblB type.

Supplementary Table 1: The sequences of forward and backward primers of the *MVK-MMAB* mutations.

SNP	Primer sequence	Annealing temperature	PCR product	Enzyme
MVK				
rs3759387	TACCACGGCATGTTCTCCAT	61°C	626bp	*HpyCH4*V
	GGCGTACTGGGTGTTTTCTG			
rs877710	AATATACCTGGCCCTCCTGC	61°C	699bp	*BstX2*I
	GGGGAAGGATAGCTGCAGAT			
MMAB				
rs9593	TCTCTCTGCCCCTCCAGACT	61°C	390bp	*Fae*I
	TGAGAAGGAGCTGTGCGGAA			
rs7134594	CCCCTAGTGCTGTCTTGTGA	61°C	481bp	*Bse1*I
	CCAGACGGTTCCTCTTGACT			
